# Supplementary material for: Binding and transport of D-aspartate by the glutamate transporter homolog GltTk
Source: eLife. 2019 Apr 10;8:e45286. doi: 10.7554/eLife.45286 (PMC6482001; doi:10.7554/eLife.45286)
Supplement: Figure 1—source data 1. — Proteoliposomes were loaded with 20 mM HEPES/Tris, pH 7.5, 200 mM NaCl, 10 μM L- or D-aspartate, 50 mM KCl and diluted 20 fold in buffer containing 20 mM HEPES/Tris, pH 7.5, 200 mM NaCl, 35.0/26.4/19.2 mM CholineCl, 0/11.1/18.4 mM KCl in the presence of 3 µM valinomycin. [file elife-45286-fig1-data1.docx]

| ΔΨ (mV) | NaCl_in_ (mM) | NaCl_out_  (mM) | D-Asp_in_  (mM) | D-Asp_out_  (mM) | KCl_in_  (mM) | KCl_out_  (mM) | CholineCl  (mM) |
| --- | --- | --- | --- | --- | --- | --- | --- |
| -78.06 | 200 | 200 | 0.01 | 0.0005 | 50 | 2.47 | 35.03 |
| -39.03 | 200 | 200 | 0.01 | 0.0005 | 50 | 11.12 | 26.38 |
| -26.02 | 200 | 200 | 0.01 | 0.0005 | 50 | 18.36 | 19.15 |
